# Validation of a self‐completed Dystonia Non‐Motor Symptoms Questionnaire

**DOI:** 10.1002/acn3.50900

**Published:** 2019-09-27

**Authors:** Lisa Klingelhoefer, Kallol R. Chaudhuri, Christoph Kamm, Pablo Martinez‐Martin, Kailash Bhatia, Anna Sauerbier, Maximilian Kaiser, Carmen Rodriguez‐Blazquez, Bettina Balint, Robert Untucht, Lynsey J. Hall, Lauritz Mildenstein, Miriam Wienecke, Davide Martino, Olaf Gregor, Alexander Storch, Heinz Reichmann

**Affiliations:** ^1^ Department of Neurology Technical University Dresden Dresden Germany; ^2^ National Parkinson Foundation International Centre of Excellence Department of Neurology King’s College Hospital London United Kingdom; ^3^ Department of Neurology University of Rostock Rostock Germany; ^4^ German Centre for Neurodegenerative Diseases (DZNE) Rostock/Greifswald Rostock Germany; ^5^ National Centre of Epidemiology and CIBERNED Carlos III Institute of Health Madrid Spain; ^6^ Sobell Department of Motor Neuroscience and Movement Disorders UCL Institute of Neurology London United Kingdom; ^7^ Department of Neurology University Hospital Heidelberg Germany; ^8^ Department of Clinical Neurosciences University of Calgary & Hotchkiss Brain Institute Calgary Canada; ^9^ Department of Neurology Klinikum Chemnitz Chemnitz Germany

## Abstract

**Objective:**

To develop and validate a novel 14‐item self‐completed questionnaire (in English and German) enquiring about the presence of non‐motor symptoms (NMS) during the past month in patients with craniocervical dystonia in an international multicenter study.

**Methods:**

The Dystonia Non‐Motor Symptoms Questionnaire (DNMSQuest) covers seven domains including sleep, autonomic symptoms, fatigue, emotional well‐being, stigma, activities of daily living, sensory symptoms. The feasibility and clinimetric attributes were analyzed.

**Results:**

Data from 194 patients with CD (65.6% female, mean age 58.96 ± 12.17 years, duration of disease 11.95 ± 9.40 years) and 102 age‐ and sex‐matched healthy controls (66.7% female, mean age 55.67 ± 17.62 years) were collected from centres in Germany and the UK. The median total NMS score in CD patients was 5 (interquartile range 3–7), significantly higher than in healthy controls with 1 (interquartile range 0.75–2.25) (*P* < 0.001, Mann–Whitney *U*‐test). Evidence for intercorrelation and convergent validity is shown by moderate to high correlations of total DNMSQuest score with motor symptom severity (TWSTRS: *r*_s_ = 0.61), clinical global impression (*r*_s_ = 0.40), and health‐related quality of life measures: CDQ‐24 (*r*_s_ = 0.74), EQ‐5D index (*r*_s_ = −0.59), and scale (*r*_s_ = −0.49) (all *P* < 0.001). Data quality and acceptability was very satisfactory.

**Interpretation:**

The DNMSQuest, a patient self‐completed questionnaire for NMS assessment in CD patients, appears robust, reproducible, and valid in clinical practice showing a tangible impact of NMS on quality of life in CD. As there is no specific, comprehensive, validated tool to assess the burden of NMS in dystonia, the DNMSQuest can bridge this gap and could easily be integrated into clinical practice.

## Introduction

Cervical dystonia (CD) is the most common idiopathic focal dystonia.^1^, ^2^ Currently, there is no CD‐specific validated questionnaire for the holistic assessment of non‐motor symptoms (NMS) in CD. Validated disease‐specific questionnaires like the Craniocervical Dystonia Questionnaire (CDQ‐24)^3^ or the Toronto Western Spasmodic Torticollis Rating Scale (TWSTRS/‐2)^4^, ^5^ assess a few NMS in a detailed disease‐specific manner whereas symptom‐specific questionnaires like Beck Depression Inventory (BDI) assess only a single NMS in a very detailed manner, with lack of knowledge of their reliability and validity in CD. Moreover some well‐described NMS in CD, for example sleep disturbances are not assessed by any of the established disease‐specific validated tools. In order to fully capture the motor and non‐motor health of a patient with CD within the short time frame of a clinical consultation, there is a need for a patient self‐completed, validated, disease‐specific NMS questionnaire similar to the one that has been developed for Parkinson’s disease (PD).^6^ CD also affects health‐related quality of life (HRQoL) in a manner which is comparable to that seen in other chronic neurological diseases like multiple sclerosis, PD, and stroke^7^, ^8^ and in part this could be driven by the burden of NMS which is currently not captured in the clinic. The objective of the present study was to provide an easy to use and clinically feasible, validated, disease‐specific NMS questionnaire for the use in patients with CD to meet this unmet need and improve the holistic assessment of CD patients.

## Patients and Methods

This was an international, multicenter, open, one point‐in‐time evaluation in English and German language with test–retest assessment. The DNMSQuest in German language was developed based on the international recognized standards for intercultural adaptation of self‐completed patient questionnaires.^9^, ^10^

### Study participants

#### Patients and controls

Consecutive patients with a diagnosis of adult‐onset idiopathic dystonia manifesting in the cervical region (cervical dystonia, CD) according to the recently revised definition by Albanese et al.^1^ were included in this study. About 97.4% of the included patients were treated with botulinum neurotoxin (BoNT) injections. To avoid the potential effect of BoNT therapy on study assessments, study inclusion was performed at the end of a BoNT therapy cycle (≥3 months after the previous BoNT treatment session) and just before a new BoNT treatment was performed. As NMS are prevalent in the general population, age‐ and sex‐matched healthy controls (HC) were also included in the study.

#### Inclusion and exclusion criteria

Patients with CD and HC were included in the study if no history of dementia or evidence of significant cognitive impairment (<22 points on the Montreal Cognitive Assessment (MoCA)^11^, ^12^) as well as no presence or history of any movement disorder other than dystonia and associated tremor was reported. Patients with other forms of idiopathic dystonia as well as patients treated with deep brain stimulation were excluded.

#### Recruitment centres

Patients were recruited from five different specialist Movement Disorders Clinics in Germany and the United Kingdom: Department of Neurology, Technical University Dresden, Germany (TUD); Department of Neurology, Klinikum Chemnitz, Germany; Department of Neurology, University of Rostock, Germany; Department of Neurology, King's College London, UK; Department of Neurology, University College London, UK. HC were recruited at the Department of Neurology, Technical University Dresden, Germany and at the Department of Neurology, King's College London, UK.

### Study assessments

The study protocol of the DNMSQuest study was approved by the ethics committee of the participant institutions (Dresden and Chemnitz: EK60022015, Rostock: A2016‐0159, King's College London: 15/EM/0106, University College London: 15/0445‐162452). All participants provided written informed consent before any study procedure was carried out.

### Dystonia Non‐Motor Symptoms Questionnaire (DNMSQuest)

**Figure 1:**
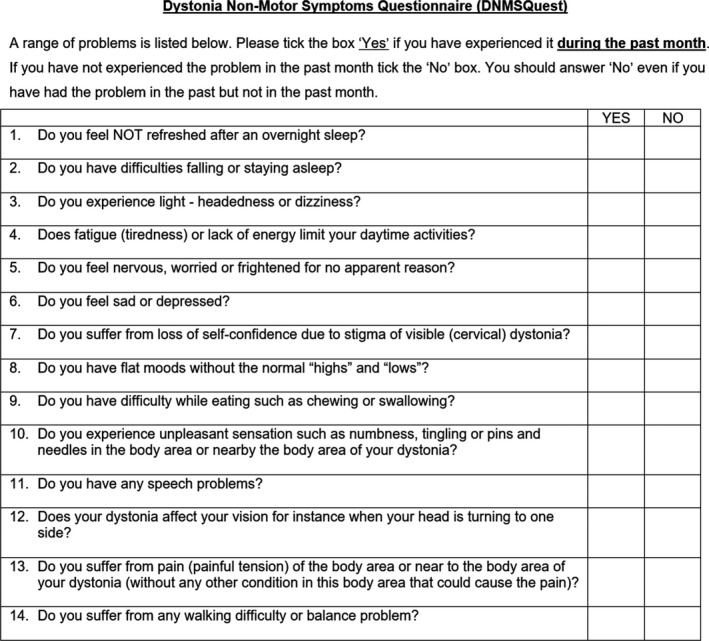
The Dystonia Non‐Motor Symptoms Questionnaire (DNMSQuest) for cervical dystonia in English language.

The Dystonia Non‐Motor Symptoms Questionnaire (DNMSQuest, Fig 1, Fig. S1) is a 14‐item self‐completed questionnaire asking about the presence of a range of NMS in patients with craniocervical dystonia during the past month. Wording of the questionnaire is adapted to lay expressions and the response option is a binary one as “yes” or “no” to each question. Completion time by patients with the aid of their caregivers, if necessary, is around 5 min. The instructions considering the time frame are outlined on the questionnaire. By summating all positive answers (“yes”, corresponding to the presence of NMS), the total number of NMS experienced by the participant can be calculated as an estimation of the symptoms burden, although any information on symptoms severity or frequency is not obtained. The questions can be grouped into seven different relevant non‐motor domains (Table 1): sleep (2 items), autonomic symptoms (1 item), fatigue (1 item), emotional well‐being (3 items), stigma (1 item), activities of daily living (ADL, 4 items), and sensory symptoms (2 items).

**Table 1:** Domains of the DNMSQuest.

Domains of the DNMSQuest	Number of DNMSQuest question	Presence of NMS (%)		*P*‐value (Fisher‐test)
		Patients with cervical dystonia	Healthy controls	
Sleep (sleep quality, insomnia)	1, 2	69.1	51.9	<0.001
Autonomic symptoms	3	30.4	11.8	<0.001
Fatigue	4	47.9	21.6	<0.001
Emotional well‐being (anxiety, depression)	5, 6, 8	52.0	28.4	<0.001
Stigma	7	50.5	1.0	<0.001
Activities of daily living (chewing/swallowing, speech, vision, balance/walking)	9, 11, 12, 14	56.7	17.7	<0.001
Sensory symptoms (paraesthesia, pain)	10, 13	75.3	13.8	<0.001

The following variables were collected:

Investigator completed:

demographic and disease‐specific clinical variables like age, sex, ethnicity, year of diagnosis, general medical history with current medication and cumulative BoNT dosage (calculated in Dysport mouse units) as well as duration of positive effects.
motor assessment: TWSTRS.^5^ Unified Dystonia Rating Scale (UDRS)^13^ with maximal possible total score of 24 as only patients with focal CD affecting the neck and shoulder or the proximal arm domain have been included.
Clinical Global Impression of severity (CGI‐S)^14^ focused on the severity of CD
MoCA.^15^

Partient‐completed assessments:

DNMSQuest.^16^, ^17^, ^18^ Quality of life questionnaires: CDQ‐24;^3^ EuroQol five dimensions questionnaire (EQ‐5D) with index and visual analog scale (VAS).^19^

Test–retest analysis: a re‐assessment of CD patients 7–14 days after initial study inclusion and after BoNT injection was performed.

Data monitoring: Data quality was constantly checked during data collection and all missing data were noted and subsequently collected after specific requests based on query lists provided to the different medical centers by TUD.

Data Availability Statement: For the kind of performed study, relevant information about the collected data and the methods used to conduct the research are fully presented in this article.

### Statistics

As data were mostly not normally distributed (tested with K.‐S.‐Lilliefors‐test), nonparametric statistics were primarily applied. Prevalence of each NMS was calculated on the sample of CD patients and HC determining the percentage of positive responses. DNMSQuest domains and total number of NMS was also computed by the sum of answers.

Data quality and acceptability: missing and fully computable data were calculated, with an acceptable criterion of < 5% of missing data.^20^

Construct validity: the convergent validity (correlation with other measures, which assess the same or similar constructs) and the discriminative validity (ability of the scale to differentiate known‐groups) were the components tested in this study. Spearman’s rank correlation coefficients were considered: “weak” if the *r*_s_‐value was < 0.3, “moderate” if 0.3–0.59, “high” if> 0.60.^21^, ^22^ Moderate to high correlations of the DNMSQuest and its domains were expected with the CDQ‐24 and its corresponding domains as well as with corresponding domains of the TWSTRS. Multiple testing corrections were performed by Bonferroni adjustment. The discriminative validity was explored by the differences in the DNMSQuest total score in CD patients and HC and in the sample grouped by CGI. For discriminative validity, Mann–Whitney and Kruskal–Wallis tests were applied. A *P*‐value of less than 0.05 was considered to indicate statistical significance.

Test–retest reliability (intra‐class correlation coefficient (ICC); 1‐way, random effect) was assessed in a subset of CD patients (*N* = 14) who repeated the DNMSQuest within 7–14 days after initial assessment. ICC values higher than 0.70 are considered acceptable.^23^

## Results

Data from 194 patients with CD and 102 age‐ and sex‐matched HC were analyzed. Demographics and disease‐specific characteristics as well as therapy are summarized in Table 2. Cognitive assessment in CD patients and HC were within normal ranges (mean MoCA value ± SD: 26.90 ± 2.37 in CD patients, 28.54 ± 1.61 in healthy controls) so that the results of self‐completed questionnaires and scores were considered to be reliable for analysis. Regarding data quality, no relevant data of the included study participants were missing and only five (4.67%) HC and 27 (12.22%) patients were excluded from data analyses due to relevant neurological co‐morbidities, MOCA < 22 points, segmental or multifocal dystonia. Only five (2.6%) CD patients were not treated with BoNT therapy. Study assessments were carried out at 13.74 ± 2.20 (mean ± SD) weeks after the last BoNT injection so as to effectively exclude influence of BoNT on motor, non‐motor, and quality of life assessments.

**Table 2:** Demographics, disease‐specific characteristics, and therapy of patients with cervical dystonia and healthy controls.

	Patients with cervical dystonia	Healthy controls	*P*‐value
Total number (*N*)	194	102	
% of female (*N*)	65.6 (127)	66.7 (68)	0.84^1^
Age (mean ± SD, yrs)	58.96 ± 12.17	55.67 ± 17.62	0.45^2^
Age range: minimum–maximum (yrs)	25.30–87.09	21.58–87.48	
Ethnicity: white/black/others (%)	97.9/0.5/1.5	83.3/7.8/8.8	
Disease duration (mean ± SD, years)	11.95 ± 9.40		
Duration of BoNT therapy (mean ± SD, years)	9.39 ± 7.43		
Total dose of BoNT (mean ± SD, Dysport MU) per treatment session	601 ± 291		
Range: minimum–maximum (Dysport MU)	40–1800		

^1^ Chi‐Squared test.

^2^ MWU test.

CD patients ranged from CGI score 1 to 7 with 81% of patients presenting with “mildly ill” (score 3) to “markedly ill” (score 5) (mean ± SD: 3.81 ± 1.16; median ± SE: 4.00 ± 0.08) severity of CD which corresponded well to a mean TWSTRS score of 31.17 ± 12.78 (range 6.0–65.5) and mean UDRS score of 7.89 ± 3.83 (range 1.0–24.0). In contrast, 92% of the assessed HC were “normal, not at all ill” (score 1) in the CGI (range 1–3; 47% of HC evaluated, 53% not assessed, mean ± SD: 0.53 ± 0.64; median ± SE: 0.00 ± 0.06).

**Figure 2:**
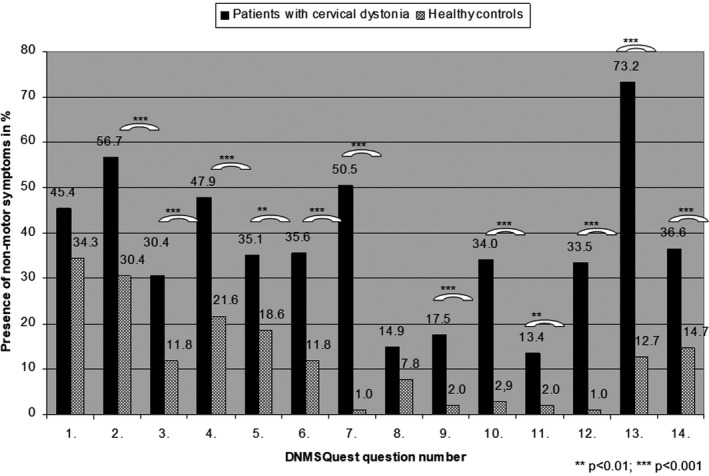
Presence of non‐motor symptoms in percentage assessed by DNMSQuest in patients with cervical dystonia and healthy controls (Chi‐Squared test; *P*‐values ** <0.01, *** <0.001).

NMS were significantly more common in CD patients compared with HC (Fig 2). Median total NMS score was significantly higher with 5.00 (interquartile rank 3–7, 23 patients, 11.9%, mean ± SD: 5.26 ± 3.25) in CD patients in comparison to 1.00 (interquartile rank 0.75–2.25, 29 patients, 28.4%, mean ± SD: 1.73 ± 1.67) in HC (*P* < 0.001, Mann–Whitney‐*U*‐test). Total NMS scores in CD patients ranged from 0 (11 patients, 5.7%) to 14 (1 patient, 0.5%), while in HC NMS scores ranged from 0 (25 controls, 24.5%) to 9 (1 control, 1.0%). About 94.3% (183) CD patients reported at least one NMS and therefore only 5.7% (11) did not suffer from any declared NMS. 54.7% (106/194) of the CD patients presented with at least five NMS, which is regarded as a mild to moderate burden of NMS in PD patients. Specifically, CD patients presented significantly more often complaints about pain, sleep disturbances, loss of self‐confidence, fatigue, walking/balance problems, mood disturbances, unpleasant sensations, and disturbed vision than HC (Fig. 2). The results of the seven DNMSQuest domains are summarized in Table 1 comparing CD patients and HC.

There was no significant association of total NMS score assessed with DNMSQuest in CD patients with age and disease duration but a significant weak correlation with sex (*r*_s_ = 0.18; *P* = 0.01). This was supported by a significant higher mean number of NMS in female than male CD patients (*P* < 0.05) (Table 3). In HC no significant association of total NMS score with age and sex was found.

**Table 3:** Known‐groups validity of the DNMSQuest total score in patients with cervical dystonia.

	*N*	Mean	Standard‐deviation	Minimum	Maximum	*P*‐value
Gender						0.014^1^
Male	67	4.52	3.36	0	14	
Female	127	5.65	3.13	0	13	
Age (quartiles)						0.948^2^
<=49	38	5.34	3.33	0	12	
50–58	64	5.44	3.24	0	14	
59–68	45	5.24	3.64	0	12	
69+	47	4.96	2.84	0	13	
CGI grouped						<0.001^2^
Normal (1–2)	20	3.15	2.89	0	9	
Mild (3)	60	4.00	2.46	0	12	
Moderate (4)	66	5.94	3.14	0	12	
Severe (5–7)	48	6.77	3.43	0	14	

^1^ Wilcoxon test.

^2^ Kruskal–Wallis test.

The total NMS score of the DNMSQuest in CD patients significantly correlated with motor severity assessed by TWSTRS total score on a high level (*r*_s_ = 0.61; *P* < 0.001) and UDRS on a weak level (*r*_s_ = 0.26; *P* < 0.001). Furthermore, we found a significant moderate correlation between the total number of NMS and CGI (*r*_s_ = 0.40; *P* < 0.001) in CD patients, whereas there was no correlation in healthy controls. Additionally, the analysis of known‐groups validity showed a significant increase in mean DNMSQuest total score with higher values in CGI (*P* < 0.001) (Table 3). The total amount of NMS measured by DNMSQuest in CD patients correlated significantly with both measures of HRQoL, the CDQ‐24 total score on a high level (*r*_s_ = 0.74; *P* < 0.001), as well as negatively on a moderate to high level with the EQ‐5D index (*r*_s_ = −0.59; *P* < 0.01) and EQ‐5D VAS (*r*_s_ = −0.49; *P* < 0.001), respectively. In 48 HC, the EQ‐5D index and VAS have been assessed. Here the total NMS score showed a significant negative correlation on a moderate level with the EQ‐5D index (*r*_s_ = −0.58; *P* < 0.001) and EQ‐5D VAS (*r*_s_ = −0.44; *P* < 0.01), respectively.

All DNMSQuest domains, besides stigma (*r*_s_ = 0.57; *P* < 0.001) and autonomic symptoms (*r*_s_ = 0.43; *P* < 0.001), correlated significantly on a high level with the DNMSQuest total score (*r*_s_ = 0.60–0.74; *P* < 0.001). Eight out of 21 correlations of the seven DNMSQuest domains among each other showed significant moderate correlations (*r*_s_ = 0.30–0.49; *P* < 0.01) (Table 4).

**Table 4:** Intercorrelation and convergent validity of DNMSQuest domains in patients with cervical dystonia.

DNMSQuest domains	Sleep	Autonomic	Fatigue	Emotional well‐being	Stigma	Activities of daily living	Sensory symptoms
Autonomic symptoms	0.27**						
Fatigue	0.32**	0.29**					
Emotional well‐being	0.34**	0.18*	0.37**				
Stigma	0.22**	0.18*	0.25**	0.39**			
Activities of daily living	0.24**	0.28**	0.43**	0.30**	0.35**		
Sensory symptoms	0.20**	0.09	0.25**	0.25**	0.28**	0.49**	
TWSTRS						part II 0.57***	
						part II question B 0.44***	
							part III 0.63***
CDQ 24				0.60***	0.64***	0.62***	Pain 0.66***

Spearman’s rank correlation coefficients.

* *P* < 0.05.

** *P* < 0.01.

*** *P* < 0.001.

As expected, correlation coefficients between corresponding domains of the DNMSQuest and related scales and questionnaires (TWSTRS, CDQ‐24) reached r_s_ values between 0.44 and 0.66 (*P* < 0.001) (Table 4).

Of the 55 CD patients performing a retest of the DNMSQuest, only 14 were obtained in the time frame of up to 14 days after initial assessment to exclude effect of BoNT therapy. Mean as well as median DNMSQuest total scores and its range were not significantly different (*P* = 0.32) when comparing initial study assessment and retest (Table 5). The ICC value with 0.995 was very high.

**Table 5:** Test–retest reliability of the DNMSQuest in patients with cervical dystonia.

	Study assessment *N* = 14	Retest *N* = 14	*P*‐value^1^
Duration between study and Re‐Test assessments (mean ± SD, days)	8.29 ± 1.14		
Range: minimum–maximum (days)	6–11		
DNMSQuest total score			
Mean ± SD	5.93 ± 3.54	6.07 ± 3.77	0.32
Median ± SE; interquartile rank	5.00 ± 0.95; 3.00–9.25	5.00 ± 1.01; 3.00–9.25	
Range: minimum–maximum	0 (7.1%) to 11 (14.3%)	0 (7.1%) to 12 (14.3%)	

^1^ Wilcoxon test.

## Discussion

We describe the development and validation of a novel, patient self‐completed, 14‐item “yes/no” questionnaire for the comprehensive assessment of NMS in CD patients in daily clinical practice. The Dystonia Non‐Motor Symptoms Questionnaire (DNMSQuest) enquires about the presence of a range of NMS in patients with craniocervical dystonia during the past month with a completion time by patients of approximately 5 min. By summating all positive answers, the total number of NMS experienced can be calculated as an estimation of the symptoms burden, as has been validated in PD.^24^ Furthermore, the DNMSQuest covers seven relevant non‐motor domains: sleep, autonomic symptoms, fatigue, emotional well‐being, stigma, ADL, sensory symptoms. The specific content and format of the DNMSQuest were developed from an international advisory group of six neurologists expert in movement disorders and dystonia, three health professionals expert in research on health measures development and validation and also patient experience with CD attending regional dystonia clinics in the UK.^16^ The items were derived partly from the NMSQuest, validated for use in PD.^6^ An observational pilot study including 102 CD patients was carried out in order to check the feasibility and acceptability of the DNMSQuest at King's College London.^17^, ^18^ Based on these results, the DNMSQuest was slightly adapted for better practical use and translated into German language by a cross‐cultural adaptation procedure. This is the first study providing information on the clinimetric attributes of the DNMSQuest with data assessed in 194 CD patients and 102 age‐ and sex‐matched HC in an international setting of two countries, Germany and the UK, and thereby providing the validation of the DNMSQuest in English and German language.

The questionnaire appears robust, reproducible, and has acceptable clinimetrics. Data quality was very satisfactory with all included participants being fully computable. Reliable responses of self‐completed questionnaires were secured by regular results in the cognitive assessment. Effects of BoNT therapy on study assessments can be largely excluded as participants were included in mean 13.74 weeks after the last BoNT injection, which was in compliance with the aim to carry out study assessments ≥ 3 months after the previous BoNT treatment session ensuring a maximum possible interval. Additionally, study participants reported a much shorter mean positive effect of BoNT therapy of 9.06 weeks than expected (13 weeks^25^). Our approach seems to be successful as no correlation of total NMS score with the duration of received BoNT therapy, with the duration between the last BoNT injection and study assessment, and with the total amount of dispensed BoNT was found. Only a weak, but significant negative correlation between the total number of NMS and the duration of a positive effect of BoNT on CD was detected (*r*_s_ = −0.21; *P* < 0.01), suggesting that the shorter the positive BoNT effect lasts, the higher the amount of NMS.

The assessed study group with nearly 66% women with a mean age of 59 years can be evaluated as a representative group for patients with CD, which mainly affects women above 50 years of age.^26^, ^27^ The majority of CD patients presented with intermediate disease severity assessed by TWSTRS and UDRS with values comparable to other publications^28^, ^29^; however, patients in all stages of the disease were included based on the CGI observed in this study.

NMS were significantly more common in CD patients compared to HC. About 94.3% CD patients reported at least one NMS and 54.7% at least five NMS. In a PD setting, five NMS reported on NMSQuest are regarded as a mild to moderate burden of NMS in PD^30^, ^31^ as well as having a major impact on HRQoL.^32^ DNMSQuest total score distribution and score distribution within the seven domains covered the complete range and for the total score the difference between mean and median was low. The NMS burden in CD patients was higher with worse motor severity of CD. This was more evident when evaluating motor severity with TWSTRS than UDRS, probably reflecting the fact that the TWSTRS is specific for the assessment of CD, whereas the UDRS is designed for dystonia assessment in general. Furthermore, the TWSTRS evaluates motor severity, disability, and pain caused by CD. As there was no significant association with the amount of NMS assessed with DNMSQuest in CD patients with age and disease duration, NMS are relevant during the whole disease course independent of age. Interestingly, we observed a higher burden of NMS in female CD patients, which was also reported in a study of sex and NMS in PD^33^.

The TWSTRS is currently recommended by the Movement Disorder Society (MDS) task force on dystonia rating scales^34^ and has been used extensively in clinical, trial‐based, and phenomenological studies of CD. In our study, TWSTRS part II as well as the single question B in part II covering the ability to perform ADL were tested against the DNMSQuest domain ADL (Table 4). In addition, TWSTRS part III was tested against the DNMSQuest domain Sensory symptoms (Table 4). We also used the CDQ‐24, a validated measure of HRQoL specifically in craniocervical dystonia which is also recommended by the MDS task force on dystonia rating scales^34^ as a further measure for convergent validity. The CDQ‐24 addresses the following NMS domains: stigma, emotional well‐being, pain, and ADL, which are determinants of HRQoL in CD patients.^8^, ^35^, ^36^, ^37^ Although the CDQ‐24 is a patient‐completed questionnaire, it has neither been extensively used nor tested against other scales, which could be due to potential patient fatigue related to the length of the scale. While testing the four DNMSQuest domains (Sensory symptoms, ADL, Stigma, and Emotional well‐being) against the corresponding TWSTRS and CDQ‐24 domains, we found significant correlations, primarily on a high level, providing a good convergent validity of the DNMSQuest as hypothesized (Table 4). The similar content of the DNMSQuest domains with the independent corresponding measures explains the high correlations but also reflects that these NMS can be assessed in a simpler and brief way, which is relevant for routine assessment in clinics. The unmet need of such a dedicated, self‐completed, comprehensive NMS tool for dystonia, which can be easily integrated in clinical practice, has already been declared.^38^, ^39^, ^40^ Furthermore, these observations highlight the importance of NMS for self‐reported health state in CD patients. All DNMSQuest domains except the domain Autonomic symptoms showed a high association with the DNMSQuest total score as well as the DNMSQuest total score with corresponding total scores of validated and accepted scores (TWSTRS, CDQ‐24, EQ‐5D) for the assessment of CD patients. Therefore, the construct validity of the DNMSQuest seems to be satisfactory.

We would also like to acknowledge potential limitations of this study:

1. We were unable to test the DNMSQuest domains Sleep, Autonomic Symptoms, and Fatigue with “gold standard” measures. However, this was not performed, given the lack of validated scales for these NMS in CD, and to optimize feasibility, concentrating on specific well‐established and validated scales in CD. Small‐scale cross‐validation studies addressing the convergent validity of DNMSQuest with respect to these other NMS should be conducted to complete validation.
2. The validation of the DNMSQuest was only performed in CD. An additional evaluation is needed in patients with cranial dystonia.
3. The relatively small size of study participants performing a retest of the DNMSQuest can be considered as a limitation. However, this reflects a real‐life situation where it is difficult for patients to re‐attend the clinic in the short time frame of 2 weeks. These preliminary results could be orientating about a potential satisfactory stability of the questionnaire, and additional analyses with appropriate sample size are needed.

In summary, in this novel study highlighting a first comprehensive, brief, easy to complete and to evaluate NMS questionnaire for craniocervical dystonia, we have demonstrated that the self‐completed DNMSQuest allows for a comprehensive and rapid screening of NMS in CD. Quick and effective assessment of NMS, now known to be a major complicating co‐morbidity in dystonic conditions, can thus be achieved in the clinic using the DNMSQuest in a scenario analogous to the use of the NMSQuest in PD. We further envisage that the DNMSQuest could possibly also be used in other forms of dystonia, although specific studies will be required. Data from the use of the DNMSQuest and subsequent grading of the NMS burden (as has been validated for the NMSQuest^30^) could also add to a truly holistic assessment of CD patients and secondarily improve management strategies. This is important as some NMS improve as consequence of reduction of motor severity in CD by BoNT (39) but some NMS need specific treatment.^35^, ^41^ Furthermore, this is particularly relevant as we have also shown that there is a strong correlation of NMS assessed with DNMSQuest and CDQ‐24 as well as EQ‐5D, implying a major impact of NMS on HRQoL in CD patients as also shown by studies with focus on specific NMS as pain, depression etc.^8^, ^28^, ^35^, ^42^ In addition, we believe that the DNMSQuest would be positioned as a specific scale separate to available dystonia rating scales, which primarily focus on the anatomical topography and the severity of motor dystonia. Some of the validated rating scales ask additionally for the dependence of the severity of motor dystonia on provoking factors like actions (The Fahn‐Marsden dystonia scale^43^), about the duration of the dystonic features (TWSTRS^5^, UDRS^13^), the disability in performing ADLs (TWSTRS^5^, The Fahn‐Marsden dystonia scales^43^), the impairment of HRQoL (CDQ‐24^3^). Recently, the Comprehensive Cervical Dystonia Rating Scale^44^ was introduced which is a combination of three scales to cover motor and psychiatric features as well as QoL. These scales focus on a broad spectrum of CD with high usefulness in clinical trials but less impact on daily practice due to the length of time required for completion by the patients as well as evaluation by the physicians.

As such, a specific, self‐completed, short but comprehensive tool providing a reasonable overview of NMS burden in dystonia remained an unmet need which can be bridged by the use of the DNMSQuest. The DNMSQuest could easily be integrated into clinical practice in the majority of movement disorder clinics and might help to implement NMS assessment in addition to the indispensable assessment of motor symptoms to guide BoNT therapy. The individual evaluation of NMS burden in each patient followed by a consequent treatment approach will be useful for the majority of CD patients, especially as NMS have a major impact on HRQoL.

## Supporting information


**Figure S1**. The Dystonia Non‐Motor Symptoms Questionnaire (DNMSQuest) for cervical dystonia in German language.

## Appendix 1

### Authorship Criteria

1 = Design or conceptualization of the study;

2 = major role in the acquisition of data;

3 = analysis or interpretation of the data;

4 = drafting or revising the manuscript for intellectual content.

**Table:**

Name	Location	Role	Contri‐bution
Lisa Klingelhoefer	Department of Neurology, Technical University Dresden, Dresden, Germany	Corresponding author	1, 2, 3, 4
K Ray Chaudhuri	National Parkinson Foundation International Centre of Excellence, Department of Neurology, King’s College Hospital, Denmark Hill, London, UK	Author	1, 2, 3, 4
Christoph Kamm	Department of Neurology, University of Rostock, Rostock, Germany, and German Centre for Neurodegenerative Diseases (DZNE) Rostock/Greifswald, Rostock, Germany	Author	2, 4
Pablo Martinez‐Martin	National Centre of Epidemiology and CIBERNED, Carlos III Institute of Health, Madrid, Spain	Author	1, 3, 4
Kailash Bhatia	UCL Institute of Neurology, Sobell Department of Motor Neuroscience and Movement Disorders, London, UK	Author	2, 4
Anna Sauerbier	National Parkinson Foundation International Centre of Excellence, Department of Neurology, King’s College Hospital, Denmark Hill, London, UK	Author	2, 4
Maximilian Kaiser	Department of Neurology, Technical University Dresden, Dresden, Germany	Author	2, 3, 4
Carmen Rodriguez‐Blazquez	National Centre of Epidemiology and CIBERNED, Carlos III Institute of Health, Madrid, Spain	Author	3, 4
Bettina Balint	UCL Institute of Neurology, Sobell Department of Motor Neuroscience and Movement Disorders, London, UK	Author	2, 4
Robert Untucht	Department of Neurology, Technical University Dresden, Dresden, Germany	Author	2, 4
Lynsey Hall	National Parkinson Foundation International Centre of Excellence, Department of Neurology, King’s College Hospital, Denmark Hill, London, UK	Author	2, 4
Lauritz Mildenstein	Department of Neurology, University of Rostock, Rostock, Germany	Author	2, 4
Miriam Wienecke	Department of Neurology, Technical University Dresden, Dresden, Germany	Author	2, 4
Davide Martino	Department of Clinical Neurosciences, University of Calgary & Hotchkiss Brain Institute, Calgary, Canada	Author	1, 4
Olaf Gregor	Department of Neurology, Klinikum Chemnitz, Chemnitz, Germany	Author	2, 4
Alexander Storch	Department of Neurology, University of Rostock, Rostock, Germany, and German Centre for Neurodegenerative Diseases (DZNE) Rostock/Greifswald, Rostock, Germany	Author	2, 4
Heinz Reichmann	Department of Neurology, Technical University Dresden, Dresden, Germany	Author	2, 4

## Appendix 2

### Conflict of Interest

**Financial Disclosure/Conflict of Interest** concerning the research related to the manuscript: None of the authors reports a financial disclosure/conflict of interest concerning the research performed in this study.

Funding sources for this study: None.

**Financial Disclosures** of all authors (for the preceding 12 months) ‐ Information concerning all sources of financial support and funding, regardless of relationship to current manuscript:

Lisa Klingelhoefer reports academic grants from EU Horizon 2020, honoraria for lectures from Berlin Partner für Wirtschaft und Technologie GmbH and Desitin.

K Ray Chaudhuri reports: Advisory board: AbbVie, UCB, Sunovion, Pfizer, Jazz Pharma, GKC, Bial, Cynapsus, Novartis, Lobsor, Stada, Medtronic, Zambon, Profile, Sunovion. Honoraria for lectures: AbbVie, Britannia, UCB, Mundipharma, Zambon, Novartis, Boeringer Ingelheim Neuroderm, Sunovion. Investigator initiated grants: Britania Pharmaceuticals, AbbVie, UCB, GKC, Bial. Aacdemic grants: EU, IMI EU, Parkinson's UK, NIHR, PDNMG, EU (Horizon 2020), Kirby Laing Foundation, NPF, MRC.

K Ray Chaudhuri and Anna Sauerbier acknowledge independent research part funded by the National Institute for Health Research (NIHR) Biomedical Research Centre at South London and Maudsley NHS Foundation Trust and King’s College London. The views expressed are those of the author(s) and not necessarily those of the NHS, the NIHR or the Department of Health.

Christoph Kamm has received honoraria for presentations/lectures and travel grants from Merz Pharmaceuticals.

Pablo Martinez‐Martin: Honoraria: from National School of Public Health (ISCIII) and Editorial Viguera for lecturing in courses; International Parkinson and Movement Disorder Society for management of the Program on Rating Scales; Air Liquide, Abbvie, Zambon, and HM Hospitales de Madrid for advice in clinical‐epidemiological studies. License fee payments for the King’s Parkinson’s Disease Pain scale.

Kailash Bhatia: to be added.

Anna Sauerbier reports grants from Parkinson’s UK and Kirby Laing, consultancy and speaker related fees from Britannia, UCB and Bial.

Carmen Rodriguez‐Blazquez: financial support from Abbvie for attending national and international meetings.

Bettina Balint is supported by the Robert Bosch Foundation.

Robert Untucht received a grant from the Stiftung Hochschulmedizin (medical university foundation) in Dresden and discloses support by Merz Pharma and Ipsen for travel costs to conferences.

Davide Martino: Honoraria for consultancies from Allergan. Grant research support from Parkinson Association of Alberta.

Alexander Storch has received funding from the Deutsche Forschungsgemeinschaft (DFG) and the Helmholtz‐Association; received honoraria for presentations/lectures/consultancies or advisory boards from Desitin, Grünenthal, UCB, Messe Bremen, and AbbVie. He has served on the editorial boards of Stem Cells and Stem Cells International.Heinz Reichmann was acting on Advisory Boards and gave lectures and received research grants from Abbott, Abbvie, Bayer Health Care, Bial, Boehringer/Ingelheim, Brittania, Cephalon, Desitin, GSK, Lundbeck, Medtronic, Merck‐Serono, Novartis, Orion, Pfizer, TEVA, UCB Pharma, Valeant, and Zambon.

Maximilian Kaiser, Lynsey J. Hall, Lauritz Mildenstein, Miriam Wienecke and Olaf Gregor report no financial disclosures.
