# Supplementary figures and images for: Validation of a self‐completed Dystonia Non‐Motor Symptoms Questionnaire
Source: Ann Clin Transl Neurol. 2019 Sep 27;6(10):2054–65. doi: 10.1002/acn3.50900 (PMC6801169; doi:10.1002/acn3.50900)

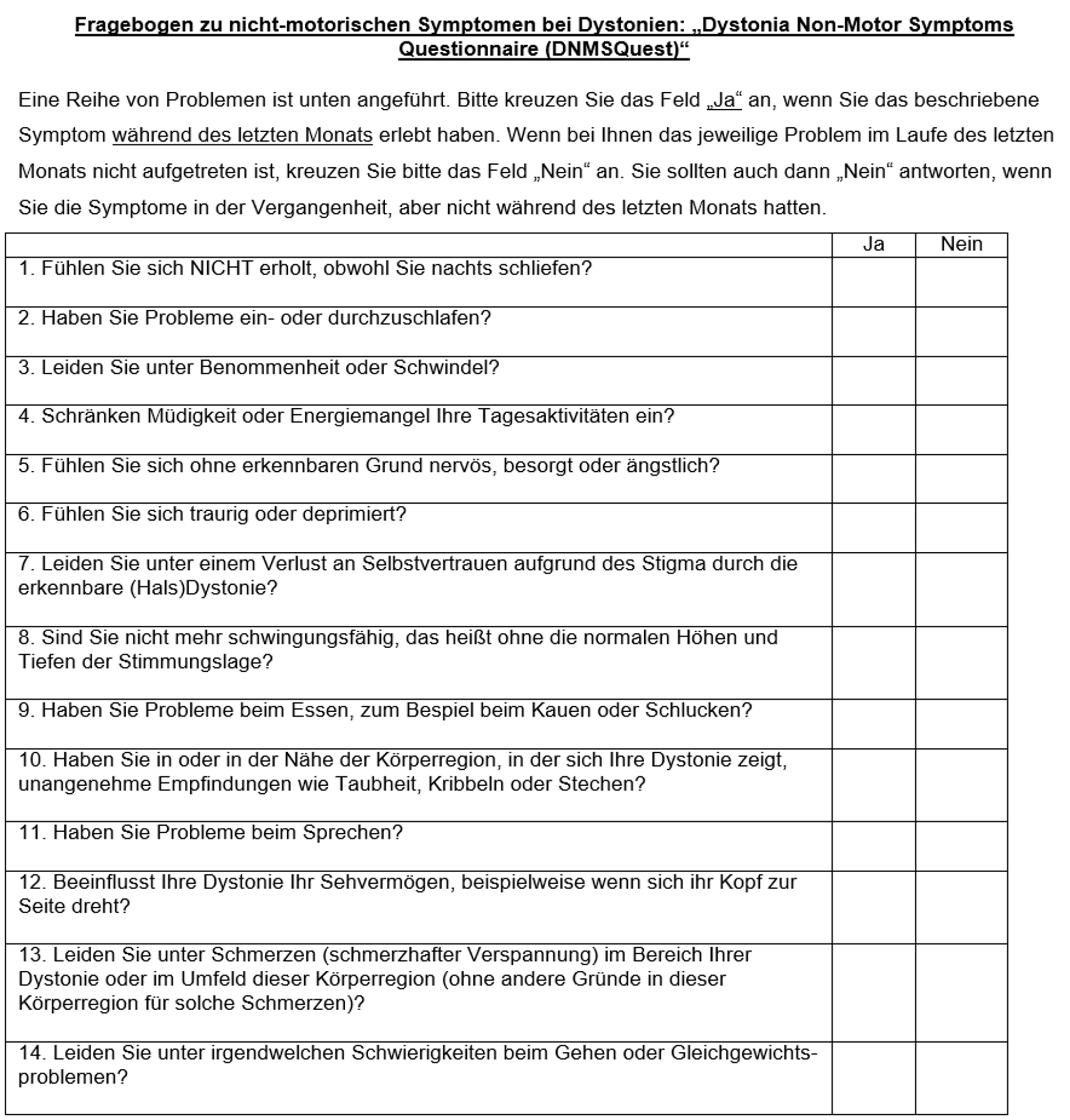

Supplement: Supplementary file 1 — Figure S1. The Dystonia Non‐Motor Symptoms Questionnaire (DNMSQuest) for cervical dystonia in German language. [file ACN3-6-2054-s001.tif]
